# Focal Task-Specific Lower Limb Dystonia Only When Walking Stairs: Is It a New Disease Entity?

**DOI:** 10.3389/fneur.2019.01081

**Published:** 2019-10-29

**Authors:** Jong Sam Baik, Hyeo-Il Ma, Phil Hyu Lee, Takaomi Taira

**Affiliations:** ^1^Department of Neurology, Sanggye Paik Hospital, Inje University College of Medicine, Seoul, South Korea; ^2^Department of Neurology, Hallym University Hospital, Anyang, South Korea; ^3^Department of Neurology, Severance Hospital, Yonsei University College of Medicine, Seoul, South Korea; ^4^Department of Neurosurgery, Neurological Institute, Tokyo Women's Medical University, Tokyo, Japan

**Keywords:** focal dystonia, walking dystonia, task-specific, lower limb, stairs

## Abstract

**Introduction:** Focal task-specific dystonia in the lower limb or foot often occurs only during walking, running, hiking, or cycling. Several medications and botulinum toxin injection are effective in patients with this disorder. The objective of this study was to understand the spectrum of focal task-specific dystonia in the lower limb only when walking stairs and to compare other types of task-specific dystonia.

**Methods:** All original articles and case reports were collected and reviewed using PubMed. In addition, all video clips of published cases were evaluated, and patients' clinical findings analyzed. The present study included 12 patients described in previous studies and five new Asian patients found in the medical records.

**Results:** Most of the patients were women, and the onset age was 42 years. Ten patients were classified as the Kicking type, including three patients with the rKicking type, and six patients were considered as the Lifting type; however, only one patient was not included in any of the types. Symptoms in most of the patients did not improve with any medications or botulinum toxin injection. The symptoms of most patients did not change over a long time.

**Conclusion:** Most patients showed the dystonic symptom when walking downstairs rather than upstairs. Psychogenic dystonia is a disease differentially diagnosed with this dystonia. Unlike other types of focal task-specific dystonia, the response to treatment was disappointing because most of the medications and botulinum toxin injection were not effective. The prognosis is completely different from that of other types of focal task-specific dystonia.

## Introduction

Dystonia is defined as a movement disorder characterized by sustained or intermittent muscle contractions causing abnormal, often repetitive movements, postures, or both (1). Lower limb dystonia is common in childhood but uncommon in adults (2–4). Adult-onset lower limb dystonia can often occur owing to secondary causes that are associated with trauma, parkinsonism, psychogenic behavior, or paroxysmal disorders. Focal task-specific dystonia (FTSD) is a well-known dystonic movement disorder that occurs only during a specific activity or task. FTSD typically occurs in the hand or face, such as writer's cramp, musician's cramp, or embouchure dystonia. Conversely, FTSD in the lower limb or foot is very rare. If patients have FTSD in the lower limb or foot, they tend to complain of their symptoms occurring during walking, running, hiking, or cycling. In addition to these intense repetitive exercises as triggering factors, in several recent case reports (5–10) and series (11), patients with unusual ambulatory characteristics as triggering factors, which occur only when walking stairs, have been reported. The authors of the present study recently encountered five patients with this unique triggering factor, and with the combination of previously reported cases, the spectrum of FTSD in the lower limb only when walking stairs was analyzed.

## Methods

### Case Collection

**Figure 1:**
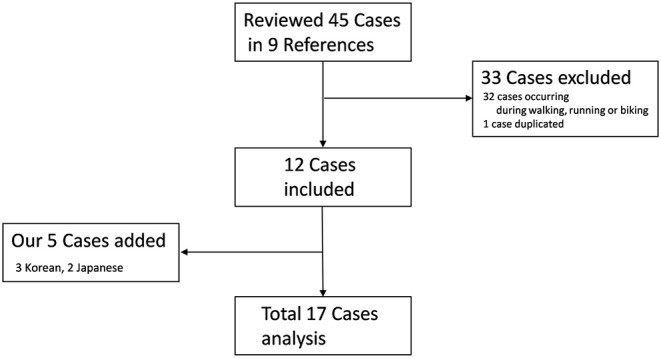
Flowchart for data collection.

Multiple searches with several key words (e.g., focal, task-specific, lower limb, foot, stairs, and dystonia) were performed, using PubMed from 2005 to 2019. All original articles and case reports were collected and reviewed, if detailed clinical manifestations were described. In addition, all video clips of published cases were evaluated, and patients' clinical findings analyzed. While examining clinical findings and reviewing the papers and video clips, the authors of case series were contacted by email for clarification of patients' clinical findings and history. After contacting several authors (5, 12), we confirmed that one case was duplicated (5, 7) and another case included a patient who experienced FTSD while not only walking downstairs but also when running and cycling (12). We found three cases in Korea of FTSD in the lower limb only when walking stairs and two cases in Japan. In the present study, inclusion criteria were the following: (A) lower limb dystonia causing foot-, knee-, or hip-related abnormal posture; (B) dystonia only when walking downstairs/upstairs; and (C) dystonia involving only a single lower limb. Patients with abnormal movements in other body parts except one lower limb and patients with dystonia triggered by intense repetitive exercise such as walking, running, or cycling in addition to walking downstairs/upstairs were excluded from the present study. Among 17 patients, 12 patients were in seven previously published cases series (5–11), and five new Asian patients (three in Korea and two in Japan) from our studies were added (Figure 1). Demographic characteristics, phenomenology, improving factors, study results, treatments, and clinical course of FTSD associated with stairs were analyzed.

### Patient Consent

Informed written consent was obtained after the patients had been given a complete description of the study using a video clip.

## Results

We reviewed seven studies on 12 patients (5–11). We also found five new patients in our medical records. Finally, we analyzed 17 patients in this study (Figure 1).

### Patient Demographics

Among 17 patients that met inclusion criteria, 13 (76%) patients were women. The mean age at initial diagnosis was 45.6 years (range, 21–67 years), and age of onset was 42 years (range, 20–56 years). The mean duration to diagnosis was 2.7 years (range, 0.7–10 years), except in two patients (patients 3 and 5) for whom the exact onset time and duration were unknown (10, 11). Patient symptoms affected both right (*n* = 9) and left (*n* = 8) sides similarly. Previous medical history showed that one patient had a calcaneus bone fracture on the same side; however, her dystonia did not occur for 2 years after trauma (patient 2) (5). In the family history of two patients, one patient's brother was born with an in-turned right foot (patient 1) (6), and the other patient's family member was diagnosed with Parkinson's disease (patient 4) (7). However, authors showed that the past and family history was not related to the patient's FTSD associated with stairs (5–7). All patients had no diurnal variation in their symptoms and no significant medical or psychiatric history (Table 1).

**Table 1:** Demographic findings of patients.

**Patient number**	**Case series (years)**	**Sex/age (years) at presentation to authors**	**Age at onset**	**Time to diagnosis (years)**	**Follow-up period**	**Past history**	**Family history**
**(Published)**							
1	Schneider et al. (6)	M/52	42	10	15	ND	Brother with in-turned foot
2	Lo and Frucht (5)	F/45	40	5	ND	Right calcaneus bone Fx—onset 2 years ago	None
3	Yamamoto et al. (10)	F/36	ND	ND	0.8	None	None
4	Ramdhani and Frucht (7)	M/31	26	5	5	ND	PD
5	Menon et al. (11)	F/67	30	≥30	ND	ND	None
6	Menon et al. (11)	F/49	46	3	ND	ND	None
7	Menon et al. (11)	F/43	40	3	ND	ND	None
8	Menon et al. (11)	M/56	56	0.7	ND	None	ND
9	Menon et al. (11)	F/34	32	1.5	ND	None	ND
10	Menon et al. (11)	F/41	39	2	ND	None	None
11	Portaro et al. (9)	F/55	53	2	ND	None	None
12	Arceri and Ratliff (8)	F/59	58	1	ND	None	None
**(Our patients)**							
13		M/56	53	3	11	None	None
14		F/54	53	1	6	None	None
15		F/54	52	2	0.5	ND	None
16		F/21	20	1	3	None	None
17		F/22	21	1	3	None	None

*ND, no data; Fx, fracture; PD, parkinson's disease*.

### Clinical Characteristics

#### Phenomenology

All patients had abnormal posture involving their single lower limb when walking downstairs/upstairs. However, the abnormal movement did not occur when walking or running on a flat surface. Fifteen of 17 patients experienced abnormal movements while walking downstairs, one patient when walking upstairs, and one patient when walking both ways. When viewing the video clip of their walking, flexion of the hip occurred in most patients when walking downstairs/upstairs. Except for one patient with abnormal movement when walking both upstairs and downstairs (patient 12), eight (50%) of 16 patients had hyperflexion at the hip level, seven (44%) had hyperextension at the knee level, and one (6%) had normal knee movement. One patient (patient 12) showed hyperflexion when walking upstairs and hyperextension of the knee when walking downstairs. Among nine patients who had flexion of the knee, including patient 12, three patients had hyperflexion of the knee during the hip extension phase. On the basis of the pattern of leg movement, patients were divided into the Kicking and Lifting types (see Supplementary Videos 1–3). The Kicking type was defined as knee extension during hip flexion, and Lifting type was knee hyperflexion with hip flexion. Three patients with backward hyperflexion of the knee during hip extension phase were classified as reverse Kicking (rKicking) type. Only one patient showed abnormal movement with dorsiflexion and external rotation only in the foot without any abnormal knee movements. On the basis of the subtypes, 10 patients (59%) were classified as the Kicking type, including three with the rKicking type, and six patients were considered the Lifting type; however, only one patient was not included in any of the types. Three patients, including one of our patients, felt that their symptom was relieved when they walked slowly while imagining that they were walking backward or on a flat surface. One of the three patients discovered that the abnormal movement improved by walking sideways downstairs (5), and another patient showed minimal improvement with the use of different shoes or by walking downstairs barefoot (7). Our patient also showed abnormal movement improvement while walking downstairs slowly while imagining normal walking (Table 2).

**Table 2:** Clinical characteristics of patients.

**Patient number**	**Case series (years)**	**Affected leg**	**Upstairs/** **downstairs**	**Phenomenology**	**Subtype**	**Improving factors**	**Investigation**	**Treatment**	**Prognosis**
**(Published)**									
1	Schneider et al. (6)	Left	Down	Hip flexion, knee extension, foot plantar flexion, internal rotation	Kicking	Walking sideways down, imagining walking steps	Image of brain, spine: normal	Trihexyphenidyl, l-dopa	NP
2	Lo and Frucht (5)	Right	Down	Hip flexion, knee hyperflexion Thigh internal rotation	rKicking	Different shoes, walking down barefoot, imagining walking backward	MRI. EMG: normal	Trihexyphenidyl, Botox	NP
3	Yamamoto et al. (10)	Right	Down	Hip flexion, knee extension, external rotation, foot external	Kicking	ND	Lumbar MRI: normal ^18^F-FDG PET: hypoperfusion of the left putamen and thalamus	Carbamazepine, clonazepam	Improved
4	Ramdhani and Frucht (7)	Right	Down	Hip flexion, knee flexion, foot plantar flexion	Lifting	ND	MRI: normal, DYT-1 (–), K-F ring (–)	Trihexyphenidyl, l-dopa, Botox	NP
5	Menon et al. (11)	Left	Down	Hip flexion, knee extension, external rotation, foot external rotation	Kicking	ND	None	Pramipexole, carbamazepine, Botox	NP
6	Menon et al. (11)	Right	Down	Hip flexion, Knee extension	Kicking	ND	None	None	ND
7	Menon et al. (11)	Left	Down	hip flexion, knee flexion, external rotation foot external rotation	Lifting	ND	MRI, EMG: normal	Tetrabenazine	NP
8	Menon et al. (11)	Left	Down	Hip hyperflexion, knee flexion, foot extension of the big toe	Lifting	ND	MRI: normal	Carbamazepine	NP
9	Menon et al. (11)	Left	Down	Hip hyperflexion, knee flexion, foot external rotation	Lifting	ND	MRI: normal	Trihexyphenidyl	NP
10	Menon et al. (11)	Right	Down	Hip hyperflexion, knee flexion, foot external rotation	Lifting	ND	MRI: normal	l-Dopa	NP
11	Portaro et al. (9)	Right	Up	Hip flexion, knee overflexion blocking	rKicking	ND	MRI, EMG, EEG: normal, DYT-1 (–) TMS: SICI 12%, SAI 15% decrease	ND	ND
12	Arceri and Ratliff (8)	Right	Up Down	Hip flexion, knee overflexion blocking Hip flexion knee extension, external rotation ankle dorsiflexion	rKicking Kicking	None	MRI: normal	ND	ND
**(Our patients)**									
13		Right	Down	Hip hyperflexion, knee flexion Foot external rotation	Lifting	Walking down slowly	MRI, EMG: normal	None	NP
14		Right	Down	Hip flexion, knee extension, external rotation foot external rotation	Kicking	None	Image of brain, spine: normal	Clonazepam	NP
15		Left	Down	Foot dorsiflexion, ext. rotation, extension of the big toe	Others	None	MRI, FP-CIT PET, SEP: normal	Clonazepam	NP
16		Left	Down	knee extension, foot plantar flexion	Kicking	None	MRI: normal	None	NP
17		Left	Down	Knee extension, foot plantar flexion, internal rotation	Kicking	None	MRI: normal	None	NP

*ND, no data; NP, no progression; K-F, Kayser–Fleischer; MRI, magnetic resonance imaging; EMG, electromyography; EEG, electroencephalography; TMS, transcranial magnetic stimulation; SICI, short-latency intracortical inhibition; SAI, short-latency afferent inhibition; FP CIT-PET, fluoro-propyl-carbomethoxy-iodophenyl-tropane positron emission tomography; ^18^F-FDG PET, [^18^F]fluoro-2-deoxy-d-glucose positron emission tomography; SEP, sensory evoked potential; l-dopa, levodopa*.

#### Investigation

Fifteen patients underwent imaging studies, including magnetic resonance imaging (MRI) of the brain or spine, fluoro-propyl-carbomethoxy-iodophenyl-tropane (FP CIT) and [^18^F]fluoro-2-deoxy-d-glucose (^18^F-FDG) positron emission tomography (PET) scan, and five patients received electrophysiological tests including electromyography (EMG) or evoked potentials (EPs); however, all findings were normal except for one patient. ^18^F-FDG PET of brain showed hypoperfusion of the left putamen and thalamus compared with the same regions on the right in patient 3. Also, the other patient (patient 11) underwent transcranial magnetic stimulation (TMS) to assess short-latency intracortical inhibition (SICI) and short-latency afferent inhibition (SAI); both SICI and SAI were reduced by 12 and 15%, respectively (9). A *DYT-1* gene test was negative in three patients (Table 2).

#### Treatment

Eleven patients (65%) were treated with medication including botulinum toxin (BoNT) type A. Most medications included trihexyphenidyl, levodopa, carbamazepine, and BoNT. A dopa agonist, tetrabenazine, or clonazepam was also used. However, symptoms in most of the patients did not improve with any medications or BoNT type A injection. In three patients treated with BoNT type A injection, there was no description on which muscles they injected it. Although one patient's symptoms dramatically improved with a low dose of clonazepam, symptoms of two of our patients treated with clonazepam were not improved (Table 2).

#### Prognosis

After an average 6.2-year (range, 0.5–15 years) follow-up period, the symptoms in most of the patients were unchanged (not aggravated or improved), except for one patient (Table 2).

## Discussion

Compared with abnormal movements in other body parts, adult-onset focal lower extremity dystonia is uncommon. The prevalence of primary focal lower limb dystonia is <1% of all adult-onset primary dystonia (13). Recently, a well-documented study was performed regarding adult-onset focal lower extremity dystonia (14). The authors analyzed 36 patients presenting with monomeric lower limb dystonia including foot torsion. Among the 36 patients, 14 patients (39%) had primary lower limb dystonia without any underlying disease. Most patients (13 of 14 patients) had insidious onset, and in 10 patients, dystonic foot torsion occurred only with ambulation, including one patient with task-specific dystonia as the initial symptom. A good study of case series regarding FTSD in lower extremities associated with intense repetitive exercise was performed (15). The authors evaluated seven of their patients and 14 patients who were in previously published case series. All patients had a dystonia associated with intense exercise, such as running, walking, dancing, or cycling. The authors stated that significant improvement in function was observed in most patients by using a variety of management strategies, such as those used for FTSD of the upper limb. However, unlike in patients with FTSD associated with repetitive exercise, no medication, including BoNT, was effective in patients with FTSD associated with stairs in the present study.

All patients in the present study had symptom onset during adulthood, and the youngest was 20 years of age. The mean age of onset in patients was 42 years, which was older than in previously published FTSD associated with repetitive exercise case series (38.3 years). FTSD associated with stairs affects women more frequently than men, and the women-to-men ratio is 3.0. In FTSD associated with repetitive exercise, the number of women affected is 1.6-fold higher than that of men (15). Considering the common occurrence of focal foot dystonia after traumatic injury, 10 of 34 patients (29.4%) with adult-onset focal lower limb dystonia consistently manifested as fixed (14), and two of 21 patients (9.5%) with FTSD associated with repetitive exercise had post-traumatic focal dystonia (15). In the present analysis, only one published case with a past trauma history of the foot was found in patients with FTSD associated with stairs. However, the authors suggested that this dystonia was not post-trauma related because the patient's FTSD symptom did not occur until 2 years later (5). Two published cases in this study had a family history (one with a brother with in-turned foot and the other with a family member with Parkinson's disease); however, the authors suggested that their family history also was not related to the patient's focal dystonia (6, 7). Based on these statements, no family and no past traumatic history are related to the symptom in FTSD associated with stairs.

The Lifting (*n* = 6) and Kicking types including three rKicking types (*n* = 10) were observed, and one patient was unclassified. Except for one patient who experienced the symptom when walking upstairs, most patients had the symptom only when walking downstairs. Interestingly, one patient had the symptom when walking both ways. The dystonic patient with symptoms occurring only when walking upstairs exhibited an impairment in the swing phase at the one lower limb muscles when walking upstairs, owing to a transient movement block soon after the beginning of hip and knee joint flexion. This led to an overflexion of the knee joint, like rKicking (8, 9). The reason that most of the patients had dystonia while walking downstairs rather than upstairs was unclear. However, considering the results of possible sensorimotor integration defects (16), dystonia when walking downstairs is more complex than walking upstairs or walking on a flat surface (17, 18). Therefore, dystonia while walking downstairs may cause sensorimotor dysfunction more than walking upstairs. Sensory trick as a relieving method is a characteristic finding in patients with dystonia and includes the phenomena of both exteroceptive and interoceptive stimuli in dystonia (19). When the actual trick was performed, two patients in this study had a relieving factor with the imagination of a known sensory trick as an interoceptive stimulus (5, 7). Symptom improvement was also observed in our patient when using a similar interoceptive sensory trick of imagining walking in a different manner while walking down the steps (patient 13). Unlike task-specific dystonia in the arm, which typically affects the dominant side, the unilateral nature of this dystonia is unexplained.

When evaluating FTSD patients, other causes of dystonia must be excluded, especially of psychogenic origin. When our patient was first examined (patient 13) before knowing that FTSD associated with stairs was a type of task-specific dystonia, the patient was diagnosed to have psychogenic dystonia. However, in general, psychogenic limb dystonia in adults most often presents as fixed dystonia rather than mobile action dystonia (20). Considering the pathophysiology of sensorimotor integration disturbance, psychogenic dystonia does not typically present with sensorimotor and synaptic plasticity abnormalities (21–24). Only one study showed that the findings during the TMS procedure including SICI and SAI, which are measures of intracortical paired pulse excitability and sensorimotor intracortical inhibition, respectively, are helpful for differential diagnosis between organic and psychogenic dystonia. According to a previous TMS study, SAI was significantly reduced in organic compared with psychogenic dystonia; however, SICI has been described as abnormal in both types of dystonia (21–23). In addition to TMS, brain or spine imaging studies (including MRI, CIT-PET, and ^18^F-FDG PET), neurophysiological tests (such as EMG, electroencephalography (EEG), and genetic studies for dystonia (including DYT-1) were utilized in most patients in FTSD associated with stairs. Most of the studies were unremarkable, like the FTSD associated with repetitive exercise case series. Interestingly, one study showed hypoperfusion of the putamen and thalamus in ^18^F-FDG PET, like the findings in some patients with paroxysmal kinesigenic dyskinesia (PKD) (10).

Currently, several treatment modalities have been attempted in FTSD patients, including oral medications (e.g., anticholinergic agents, levodopa, baclofen, clonazepam, and phenytoin), BoNT type A, surgery, and physical therapy. BoNT type A injection is a commonly used treatment for FTSD. In a previous study, benzodiazepine medication and BoNT injection were the only effective treatments in FTSD associated with repetitive exercise patients (15). However, in the present FTSD associated with stairs case series, no medication or BoNT injection helped improve the dystonic symptom. Interestingly, Yamamoto et al. found that the dystonia was highly responsive to a low dose of clonazepam and that ^18^F-FDG PET showed hypoactivity in a circuit of the basal ganglia in their patients (10). On the basis of these findings, they presented that FTSD associated with stairs might be linked with PKD (10). Two of our patients were treated with clonazepam also, but they did not respond to clonazepam, although they did not undergo ^18^F-FDG PET. For this reason, additional similar cases are needed to clarify whether the FTSD associated with stairs has a mechanism like that of PKD. Taira et al. were the first to report the long-term effects of ventro-oral (Vo) thalamotomy on FTSD, especially in foot dystonia (12). The patient's dystonia during running, cycling, or walking downstairs was improved dramatically after surgery, indicating that Vo thalamotomy can be a helpful treatment in patients with FTSD associated with stairs. The prognosis in the present study was consistent (not aggravated or improved) over a long time (average follow-up, 5.5 years; range, 0.5–15 years).

In conclusion, unique and important features were found in this case series. First, like in other types of FTSD, patients with FTSD associated with stairs have symptom onset during adulthood, which occurs more often in women than men. Second, considering the pathophysiology of FTSD associated with stairs, most patients showed the dystonic symptom when walking downstairs rather than upstairs. Phenomenologically, the patient had two major features that were classified to be those of the Lifting and Kicking types, although differential associating factors between the two types were not observed. Third, psychogenic dystonia is a disease differentially diagnosed with FTSD associated with stairs. Therefore, the underlying psychological factors should be considered, and several neurological examinations should be performed to exclude psychogenic disorders. Fourth, unlike FTSD associated with repetitive exercise, the response to treatment of FTSD associated with stairs was disappointing because most of the medications and BoNT injection were not effective. Also, Vo thalamotomy was recently shown to be effective in FTSD patients who have dystonia not only when running and cycling but also when walking downstairs. Lastly, the prognosis of FTSD associated with stairs is completely different from that of other FTSD types; that is, it remained unchanged without progression over a long time, which is why patients with this type of dystonia declined further treatment. Further studies and increased knowledge will help facilitate appropriate treatment and good outcomes for patients with FTSD associated with stairs.

## Author Contributions

JB, H-IM, PL, and TT contributed to the recruitment and clinical assessment. JB conceived of the study and contributed to the analysis. H-IM, PL, and JB contributed to the review and critique of analysis and drafted the initial version of the report.

### Conflict of Interest

The authors declare that the research was conducted in the absence of any commercial or financial relationships that could be construed as a potential conflict of interest.

## Supplementary Material

Supplementary Video 1 Patient 13 is shown with right lower limb task-specific dystonia when walking downstairs. Hyperflexion at the right hip and flexion of his knee and foot external rotation are seen (Lifting type).

Supplementary Video 2 Patient 14 is shown with right lower limb task-specific dystonia when walking downstairs. Flexion at the right hip and external ration with extension of her knee, like a football kick, and foot external rotation are seen (Kicking type).

Supplementary Video 3 Patient 16 is shown with left lower limb task-specific dystonia when walking downstairs. Extension at the left knee and foot plantar flexion are seen (Kicking type).
